# Radiological Identification and Review of Lumbosacral Paraspinal Muscle Pyomyositis and Abscesses in a Young Male With Streptococcus Bacteremia: A Case Report

**DOI:** 10.7759/cureus.78478

**Published:** 2025-02-04

**Authors:** Carter Wegner, Adam Roitman, Lorena Del Pilar Bonilla, Adrian Garcia

**Affiliations:** 1 Department of Translational Medicine, Florida International University, Herbert Wertheim College of Medicine, Miami, USA; 2 Department of Translational Medicine, Florida International University, Herbert Wertheim College of Medicine, Baptist Health South Florida, Miami, USA

**Keywords:** lumbosacral paraspinal pyomyositis, magnetic resonance imaging, musculoskeletal infection, pyomyositis, streptococcus bacteremia

## Abstract

We present a case report of a 21-year-old male patient with lumbosacral pyomyositis and abscesses secondary to *Streptococcus parasanguinis*, along with a review of the literature. The patient was admitted with acute metabolic encephalopathy, rhabdomyolysis secondary to cannabinoid use, acute kidney injury, and right lower extremity weakness. T2-weighted magnetic resonance imaging (MRI) of the lumbar region demonstrated multiple ring-enhancing nodular lesions within the left multifidus muscles, left erector spinae muscle, and left quadratus lumborum muscle. He was successfully treated with ceftriaxone and discharged on cefadroxil with improved right lower extremity strength. This case highlights MRI as an integral modality for the detection and treatment guidance of pyomyositis, providing a relevant microbiological-radiological association for rare causes of pyomyositis, such as *Streptococcus parasanguinis*.

## Introduction

Pyomyositis is a bacterial infection involving the skeletal muscle with a high rate of complications, including abscess formation [1]. Staphylococcus aureus accounts for 90% of pyomyositis cases, while Group A Streptococci accounts for a lower percentage, ranging from 1% to 5% [2,3]. *Streptococcus parasanguinis *is a gram-positive, viridans streptococcus, non-sporulating facultative anaerobe, and a common human pathogen prevalent in the oral cavity. It is considered an opportunistic pathogen for infective endocarditis and a rare cause of pyomyositis [4-6]. Magnetic resonance imaging (MRI) remains an integral imaging modality, demonstrating superiority over CT and ultrasound in differentiating similarly presenting soft tissue infections such as pyomyositis, discitis, osteomyelitis, and epidural abscess. This case highlights MRI as an essential tool for the detection and treatment guidance of pyomyositis, providing a relevant microbiological-radiological association for rare causes of pyomyositis, such as *Streptococcus parasanguinis*.

## Case presentation

The patient is a 21-year-old male brought in by emergency medical services after being found unresponsive secondary to a suspected drug overdose. In the emergency department, the recorded Glasgow Coma Scale Score was 8, with mental status changing from unresponsive to awake without administration of opioid receptor antagonists or additional interventions. He had no self-reported past medical history and denied any history of tobacco, alcohol, recreational drug use, or intravenous/intramuscular drug injection use. He became agitated upon questioning with physical examination demonstrating tachycardia, tachypnea, and right lower extremity pain, paresthesia, and decreased range of motion. Several doses of lorazepam were required to manage his agitation.

Upon admission, vital signs were obtained, along with a complete blood count (CBC), basic metabolic panel (BMP) with liver function tests, magnesium, creatine phosphokinase (CPK), ionized calcium, lactic acid levels, arterial blood gas, urinalysis, and a drug screening panel, with results shown in Table 1.

**Table 1:** Patient laboratory findings with reference ranges. CPK: creatine phosphokinase, HPF: high power field.

Parameter	Patient value	Reference range
Vital signs		
Temperature	36.6°C	36.1–37.2°C
Heart rate	114 bpm	60–100 bpm
Respiratory rate	29 breaths/min	12–20 breaths/min
Blood pressure	166/145 mmHg	<120/<80 mmHg
SpO_2_ (room air)	88%	>95%
Complete blood count (CBC)		
White blood cell count (WBC)	3.796×10³/µL	4.5–11×10³/µL
Hemoglobin	16.9 g/dL	13.5–17.5 g/dL (male), 12–15.5 g/dL (female)
Hematocrit	53.9%	41–50% (male), 36–44% (female)
Platelet count	404,000 µL	150,000–400,000/µL
Basic metabolic panel (BMP)		
Potassium	5.2 mmol/L	3.5–5.0 mmol/L
BUN	26 mg/dL	7–20 mg/dL
Creatinine	2.66 mg/dL	0.6–1.2 mg/dL
Glucose	179 mg/dL	70–99 mg/dL
Total calcium	Within normal limits	8.5–10.2 mg/dL
Total protein	Within normal limits	6.4–8.3 g/dL
Albumin	Within normal limits	3.4–5.4 g/dL
Lipase	Within normal limits	0–160 U/L
Liver function tests (LFTs)		
ALT	70 U/L	7–56 U/L
AST	91 U/L	8–48 U/L
Alkaline phosphatase	170 U/L	44–147 U/L
Other tests		
Magnesium	3.3 mg/dL	1.7–2.2 mg/dL
CPK	2545 U/L	10–120 U/L
Ionized calcium	1.04 mmol/L	1.12–1.32 mmol/L
Lactic acid	6.26 mmol/L	0.5–2.0 mmol/L
Arterial blood gas (ABG)		
pH	7.2	7.35–7.45
pCO_2_	32.5 mmHg	35–45 mmHg
PaO_2_	99.2 mmHg	80–100 mmHg
Bicarbonate	12.5 mmol/L	22–28 mmol/L
Saturation	96%	>94%
Urinalysis		
Gross hematuria	Present	Absent
Leukocyte esterase	Trace	Negative
WBC/HPF	2–5	0–5/HPF
Proteinuria	Present	Negative
Urine creatinine	100 mg/dL	20–275 mg/dL
Drug screen	Positive for benzodiazepines and cannabinoids	Negative

A CT scan of the head showed no acute traumatic injury. A chest X-ray showed no evidence of pneumothorax or focal opacity. A CT scan of the neck showed no acute fracture. The patient was treated with IV fluids and admitted to intensive critical care for acute metabolic encephalopathy, rhabdomyolysis secondary to cannabinoid use, acute kidney injury, lactic acidosis, and right lower extremity weakness.

**Figure 1:**
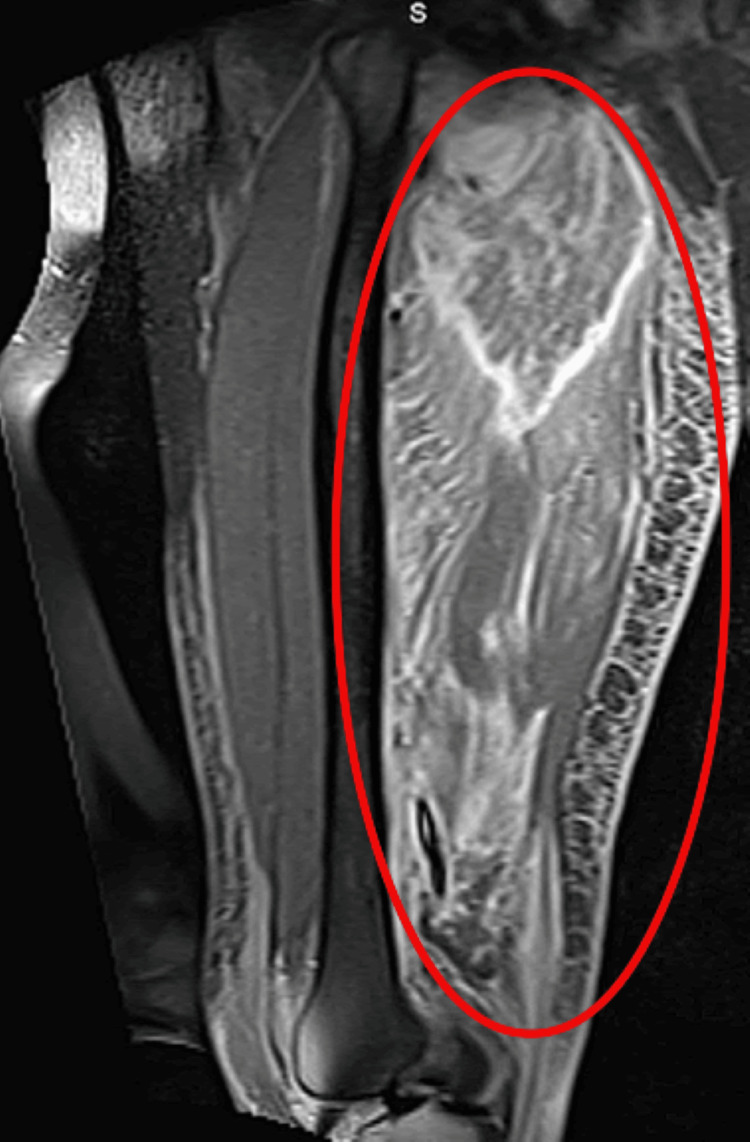
Sagittal T2-weighted MRI of the right femur demonstrating muscle edema secondary to pyomyositis. The hyperintense signal (red circle) on sagittal T2-weighted MRI of the right femur demonstrates diffuse muscle edema and inflammatory changes, suggestive of pyomyositis involving the hamstring, proximal quadriceps, and adductor muscle compartments. MRI: magnetic resonance imaging.

**Figure 2:**
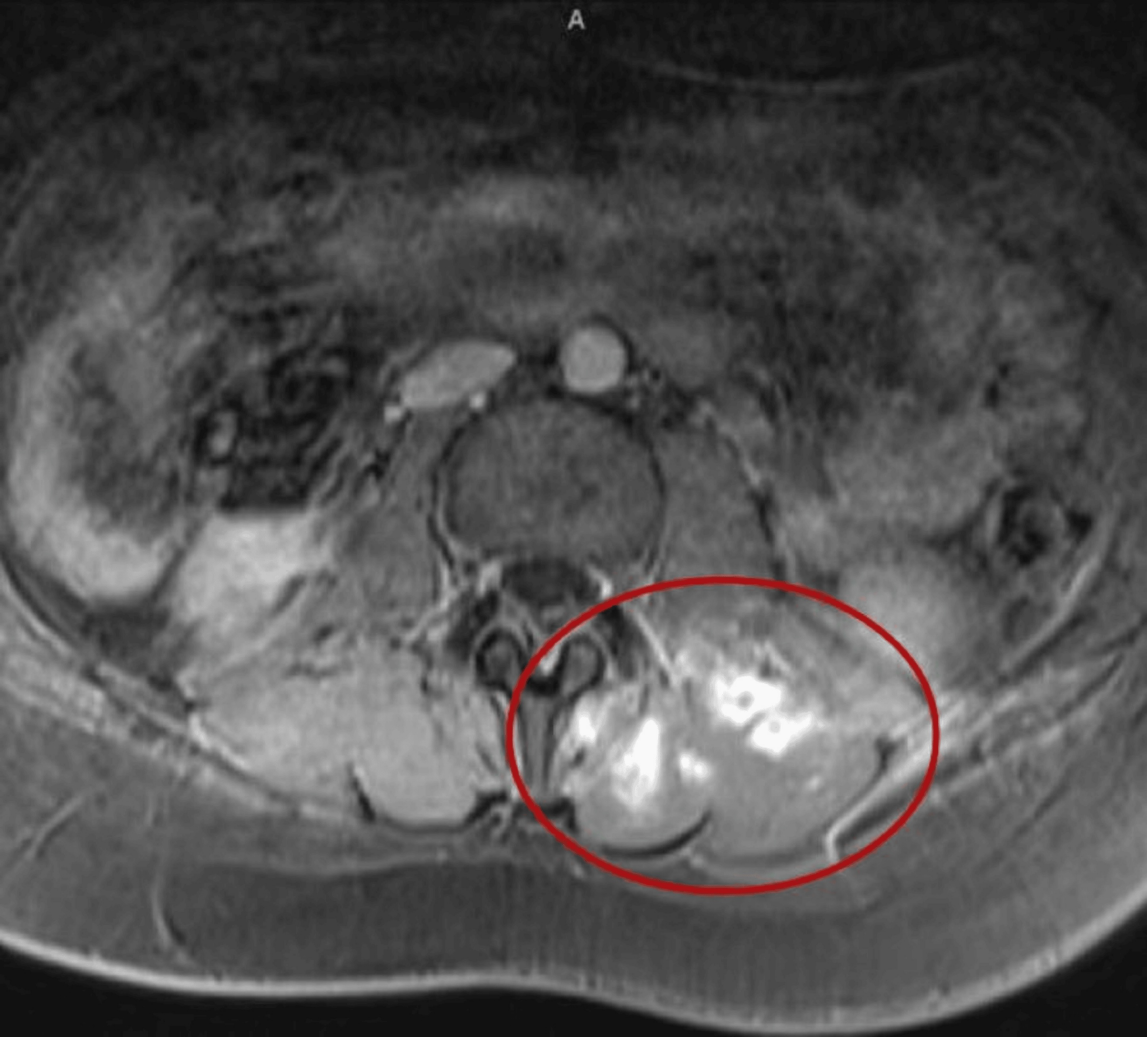
Axial T2-weighted MRI of the lumbar region showing multiple ring-enhancing nodular enhancing lesions consistent with pyomyositis. Axial T2-weighted MRI of the lumbar spine and posterior abdominal region demonstrates multiple ring-enhancing nodular lesions (highlighted within the red circle) involving the left multifidus, erector spinae, and quadratus lumborum muscles. These findings are consistent with focal areas of inflammation, edema, and soft tissue involvement, which are representative of pyomyositis. MRI: magnetic resonance imaging.

Physical examination revealed difficulty moving the right lower extremity, with the limb fixed in external rotation. There was significant difficulty with flexion at the hip joint and paresthesia of the right lower extremity. On day four of admission, a T2-weighted right femur magnetic resonance imaging (MRI) scan (Figure 1) showed extensive diffuse muscle swelling and edematous changes involving the entire hamstring, adductor muscle compartments, and proximal quadriceps. T2-weighted MRI of the lumbar region (Figure 2) revealed multiple ring-enhancing nodular lesions within the left multifidus muscles, erector spinae muscle, and quadratus lumborum muscles. Intravenous contrast was not utilized in this patient due to the presence of acute kidney injury and elevated creatinine levels. Of note, the patient did not report any previous history of blunt soft tissue injury that could contribute to these findings.

The patient was started on ceftriaxone 2 g every 24 hours. After initiation of antibiotic therapy, the patient regained strength in the right lower extremity, rated 3/5. Flexion and extension at the knee joint were regained; however, mild difficulty with hip flexion persisted. On day nine of hospitalization, a repeat lumbar MRI demonstrated near-complete resolution of the previously noted left-sided lumbosacral paraspinal muscle and intramuscular abscesses.

The patient was diagnosed with *Streptococcus parasanguinis *bacteremia, confirmed by one set of blood cultures collected on admission. Repeat blood cultures, obtained 72 hours after admission, remained sterile. The hospital stay was complicated by an acute right femoral vein deep vein thrombosis (DVT). The patient was started on enoxaparin 90 mg every 12 hours and discharged on apixaban 5 mg twice daily for three months.

Rhabdomyolysis peaked at a CPK level of 77,000. The patient’s acute kidney injury, secondary to rhabdomyolysis, resolved with hydration, and outpatient follow-up for residual proteinuria was recommended. The metabolic encephalopathy, likely secondary to drug use, also resolved.

The patient was discharged on oral cefadroxil 1 g every 12 hours, completing a 14-day course from the day of sterile cultures. Upon discharge, he was advised to follow up with hematology and was referred to a drug rehabilitation program by psychiatry, which he refused.

## Discussion

The presented case of lumbosacral paraspinal muscle pyomyositis and abscesses caused by *Streptococcus parasanguinis* highlights the critical role of radiologic imaging, particularly MRI, in the diagnosis and management of rare musculoskeletal infections. MRI was the cornerstone imaging modality, identifying diffuse muscle swelling and edematous changes of the entire hamstring, adductor muscle, and proximal quadriceps (Figure 1), along with the multiple ring-enhancing lesions within the left multifidus, erector spinae, and quadratus lumborum muscles (Figure 2). Together, MRI provided essential imaging findings to confirm, diagnose, and provide timely treatment for this case.

These findings directly align with prior studies, emphasizing the utility of MRI for early detection of muscle inflammation before abscess formation [7]. In Javed et al. (2021), paraspinal muscle inflammatory changes identified by MRI demonstrated critical findings, pivotal for differentiating bacterial pyomyositis from other conditions, such as discitis or viral myositis, that present with similar features [3].

Pannaraj et al. (2006) demonstrate the utility of MRI, and its superiority over CT and ultrasound, when evaluating soft tissue infections [1]. This is in agreement with the case findings, demonstrating the use of MRI as an effective modality for detecting inflammatory changes, abscesses, and visualizing abscess boundaries crucial for determining the extent of pathology and monitoring its resolution. Repeat lumbar MRI, for this case, displayed near-complete resolution of abscesses, validating the treatment effectiveness and the use of MRI imaging to monitor progress.

Thus, the case supports MRI as an early detection modality to prevent the progression of polymyositis and abscess formation, as evidenced by the patient’s positive response to treatment without surgical intervention. This finding supports previous studies where radiologic reassessment was integral in confirming therapeutic success and identifying the need for potential surgical drainage [1].

Limitations still exist, including the absence of specific radiologic markers for certain pathogens like *Streptococcus parasanguinis*. This underscores the importance of correlating imaging findings with clinical and microbiological data for accurate diagnosis [7]. However, it should be emphasized that MRI plays a pivotal role and demonstrates importance in diagnosis and follow-up care of such infections and should remain the modality for early pyomyositis detection and prevention of complications.

## Conclusions

The presented case of a 21-year-old male with pyomyositis and *Streptococcus parasanguinis *bacteremia highlights the critical role of MRI in the identification and management of pyomyositis. MRI demonstrated clear superiority in diagnosis, enabling timely therapeutic decisions and providing detailed visualization of pathology and its resolution to guide patient management. This case aligns with existing evidence supporting the use of MRI as the gold standard for detecting soft tissue inflammation and abscesses. Additionally, it contributes to the growing body of literature on the radiologic evaluation of pyomyositis, particularly those involving rare pathogens such as *Streptococcus parasanguinis*.
